# The association between vision acuity, sleep duration, and physical activity among US adults aged 50 years and older

**DOI:** 10.1093/geroni/igab046.3262

**Published:** 2021-12-17

**Authors:** Shu Xu

**Affiliations:** University of Masschusetts Boston, Dorchester, Massachusetts, United States

## Abstract

Studies suggested that people with low vision are more likely to have worse sleep quality and less frequent participation in physical activities compare with people with better vision. Studies also showed that physical activities is a very important factor for one’s sleep. However, there is relatively little research on the association between vision acuity, sleep, and physical activity. This study examines the relationships between vision acuity and sleep duration among middle-aged and older adults in the US, and the role of leisure-time physical activity in this relationship. Using nationally representative data from the National Health and Nutrition Examination Survey 2007-2008, a cross-sectional analysis on adults age 50 years and older was conducted (n=2.247). Visual acuity was assessed by participant’s vision of better-seeing eye (i.e., none, mild, moderate, and server visual impairment), and we measured sleep duration (i.e., short, average, and long duration) and leisure-time Physical Activity (i.e., inactive/insufficiently active and sufficiently active). Descriptive analysis showed that 31.06% of older adults experienced moderate or severe visual impairment, and 46.81% respondents experienced abnormal sleep duration. Multinomial logistic regression analyses showed that compared to people without visual impairment, people with moderate or severe visual impairment were more likely to have longer sleep duration than normal sleep duration (OR, 1.62, p<0.05). Leisure-time physical activity was not found to significantly mediate the relationship between visual acuity and sleep duration. Other variables were controlled in the models. Findings suggest that US adults age 50+ with low vision are at greater risk of experiencing abnormal sleep duration.

